# Communicating with Youth about Pain: Developmental Considerations

**DOI:** 10.3390/children7100184

**Published:** 2020-10-15

**Authors:** Natacha D. Emerson, Brenda Bursch

**Affiliations:** UCLA Department of Psychiatry, David Geffen School of Medicine, 760 Westwood Plaza, UCLA Semel 48-241, Los Angeles, CA 90024-1759, USA; bbursch@mednet.ucla.edu

**Keywords:** pain, pain communication, acute pain, procedural pain, chronic pain, pain education

## Abstract

Background: Pain experiences can negatively impact children and adolescents, leading to trauma symptoms and nonadherence to important health behaviors. Developmentally-tailored communication strategies may mitigate this risk. Methods: This article reviews cognitive and linguistic developmental factors, within the familial and cultural context, that are important to consider when communicating with youth about acute, procedural, and/or chronic pain. Results: Youth undergoing acute or procedural pain benefit from pain education, truthful information about the procedure, and advance preparation. The use of analogies may be particularly helpful for patient understanding of chronic pain development, maintenance, and treatment. Youth with developmental disabilities may express pain differently than their normative peers, requiring adaptation of communication strategies. Conclusion: Developmentally-tailored pain communication is an important tool for caregivers and healthcare providers that may foster adaptive functioning in youth who experience pain.

## 1. Communicating with Youth about Pain: Developmental Considerations

The provision of accurate, developmentally appropriate information is the first important step in helping youth cope with acute, procedural or chronic pain experiences. If successfully delivered, such pain education can reduce the distress and negative memories associated with painful events, and decrease the risk of developing iatrogenic medical trauma and nonadherence to important health behaviors [1,2]. To increase the effectiveness of pain education with youth, one must match the delivery of such information to the appropriate developmental level of the child or adolescent. Domains of child development include cognition, speech and language, social interaction, emotional regulation, physical skills, and sensory awareness. For purposes of pain-related communication, this paper will focus primarily on the domains of cognition, and speech and language development. An overview of pain communication development is then provided, highlighting ways in which intrapersonal factors (age, pain experiences) and interpersonal factors (parental modeling, cultural variables) influence children’s understanding of their own pain. The subsequent two sections summarize evidence-based strategies to optimize pain preparation through accurate and developmentally-tailored information provision. This review concludes with clinical practice parameters for supporting pediatric patients in acute and chronic pain.

## 2. Cognitive Development

Cognitive development refers to the accumulation of knowledge and thinking skills acquired as the brain develops and as the child interacts with his or her environment. Such knowledge and skills help children process information and understand the world around them. Some look to Piaget’s theory and stages of cognitive development as a helpful framework. Piaget’s four stages are sensorimotor, preoperational, concrete operational and formal operational. Although this has limitations as a model, it is presented here for reference.

In the sensorimotor stage, young children acquire knowledge through sensory experiences and by manipulating objects. At the end of the sensorimotor stage, representational thought emerges. This allows object permanence/constancy to develop and lays the foundation for language development. A major accomplishment of the preoperational stage is the emergence of language. While concrete and self-focused in their thinking, children typically become more skilled in pretend play during this stage. During the concrete operational stage, children become better at using logic in a concrete manner and start to acquire recognition that others might have thoughts, reactions or experiences that differ from their own. Children in the formal operational stage have capacity to process more complex information, including improved use of logic, use of deductive reasoning, and the ability to understand abstract ideas. They have the potential to see multiple solutions to problems and to use basic scientific methods to test out ideas [3]. Using these basic constructs, Table 1 summarizes implications for youth experiencing pain based on Piagetian cognitive level and serves as a helpful starting point for thinking about pain discussions with youth.

Clinician communication that is congruent with the cognitive abilities of the child will be most effective. In the absence of more precise information about cognitive functioning, the table above gives the clinician a place to start. Careful observation of the responses of the child will allow the clinician to alter communication as needed. For example, using the table above, a 12-year-old may have the cognitive capacity to understand that anxiety can exacerbate pain related to a tumor. However, this biopsychosocial explanation might be too complex and abstract for some 12-year-olds. The clinician will be less likely to successfully help an average 8-year-old to grasp this concept.

## 3. Pain-Related Vocabulary in Children

Communicating with youth about pain also requires caregivers and healthcare providers to grasp the ways in which pain communication develops across childhood. Pain communication is evolutionary and essential to the survival of young children who must learn to verbalize the need for assistance from caregivers in order to address illnesses and injuries common to childhood [5].

Neonates and infants communicate distress principally through crying. Early in infancy, caregivers learn to distinguish different types of cries for hunger, fatigue, and pain, each vocalization growing more distinguished as it starts to resemble speech acts [6]. The ability to communicate pain effectively is therefore a developmental process that improves over time.

As young children learn language, they refine their ability to express feelings, both physical and emotional. Young children first use simple words to denote pain, such as *hurt, ouch*, and *ow* [7]. Typically-developing children develop this basic pain language between one and two and half years of age, the description of pain related to illness developing slightly later than that used to describe injury [8].

The ability to use and comprehend the word “pain” develops later in childhood [7]. This has several implications for medical settings, in which the overreliance on having patients describe pain may confuse young patients. The use of pain faces as symbols of discomfort such as the Wong–Baker Faces Pain Rating Scale appears to reliably allow young patients to anchor their physical discomfort to a developmentally-understood cartoon face [9].

Pain communication has many other subtle developmental processes. Even after children learn to state when something hurts, the ability to describe location, type, and intensity of pain takes more time. In regard to location, pain location assessment tools may be useful for younger children who may not know how to denote the subjective source of their discomfort. Such a tool may be particularly useful for assessing post-operative pain that may be occurring outside of the immediate surgical site (e.g., neuropathic pain) [10]. Communicating type or quality of pain is even more complicated given our reliance on highly abstract, even metaphorical language such as: “It feels like my head is splitting.” The analogical nature of pain expression is difficult for young children who tend to be concrete thinkers and have fewer experiences to draw metaphors from. Further confusing to the young mind is our tendency to describe noxious stimuli as acting upon the body (e.g., shooting or stabbing) [5].

The expression of pain appears to be strongly influenced by familial and cultural factors [6]. Culturally, variations occur not only in terms of verbiage use but also in terms of frequency and acceptability of pain communication. Pain socialization appears to start early in infancy and to differ across cultures, with noted variations in both behavioral markers (e.g., behavioral reactivity, length of crying, amount of time to calming down) and physiological markers (e.g., cortisol levels) [11]. In later childhood, children of certain communal cultures have been shown to vocalize pain less as the expression of distress may be discouraged for its negative impact on others [11]. Cultures also show differences based on child gender, with some discouraging pain expression in male children [12]. Children may also moderate their own responses to pain if in the presence of someone from another culture. For instance, African American children appear to be less vocal about discomfort in front of Caucasian providers [11].

Caregivers influence their child’s pain communication both by modeling their own coping styles to negative experiences and by the way they respond to their child’s hurt. For the same scraped knee, one parent may encourage their child to “dust themselves off” and keep playing, while another may insist on stopping everything to address the wound and their child’s emotional reaction. Families also vary in their approach to pain control. Caregivers may urge children to seek social support for emotional validation (“Please come and tell Mommy when it hurts”), others may encourage the use of distraction (“Let’s play a game”), and yet other caregivers may be inadvertently invalidating of their child’s experience (“Oh come on, it’s not that bad”). In general, action-focused strategies that encourage the child to distract and function despite pain appear to be protective [1]. Familial experiences of pain can also affect the development and/or maintenance of pain. Chronic pain in one parent appears to be predictive of the development of pain conditions in their youth [13]. Besides modeling, caregivers’ own cognitions about pain can be passed on to their children. Parents who tend to catastrophize physical discomfort tend to have children who cope with pain less effectively [14]. As such, pain and its communication must be understood within the social environments in which they occur [6].

Children may vary in their communication of pain based on their audience. Children tend to emote more pain expressions in the presence of known caregivers than in the presence of strangers [15]. Likewise, children’s temperament and communication style may elicit different responses in caregivers. Children’s tendency to catastrophize versus minimize, their facial expressions of pain, and the subjective appearance of pain intensity all influence parental responses [16]. In parallel, caregiver responses, both to the immediate pain and in terms of historical pain responding, also influence the child’s pain experience [6,13]. Pain communication is thus socialized over time within familial and cultural contexts [6]. 

Pain expression also differs based on educational levels and prior pain experiences. For instance, children with chronic pain may use more emotional language to describe pain than children with no persistent pain experience [17]. As such, provision of accurate, developmentally-appropriate pain education should depend, not only on patients’ conceptual knowledge and literacy levels, but also on the degree to which children have experienced significant pain in their past [17].

## 4. Effective Caregiver and Health Provider Communication Skills

The language and communication skills of children differ from those of adults in quality and quantity. Adapting communication approaches based on these differences will yield better understanding and outcomes, especially among younger children. Those who are developmentally ten years of age or younger rarely engage in spontaneous speech with professionals. Thus, it can be helpful to possess the skills necessary to encourage the young child to engage in a discussion about pain [4].

As is true of patients of any age, it helps communication to develop rapport with the young child before trying to provide them with pain education. Minimize the expectation that demands will be placed on the child and focus on getting to know the child first. This can happen verbally or nonverbally, such as through play. Spending some time getting to know the child will allow the professional to get a sense of a child’s cognitive and linguistic developmental level to communicate with the child appropriately. Questions that work across the childhood span include: “What do you like to do for fun?” and “What do you want to be when you grow up?” Showing interest in the child outside of the provider’s assessment of pain is helpful for encouraging patient participation and cooperation. Child-directed play also has utility for assessing a child’s cognitive levels; both type and complexity of play may signal what a child is capable of grasping in terms of information provision [18]. The professional should consider that a child might have linguistic, cognitive, or both challenges if s/he appears to struggle to understand questions.

Rapport is supported by joining the young child in an activity they are doing rather than asking them to stop playing to join a discussion, whenever possible. It can be helpful to reinforce communication with praise and by demonstrating that you want to hear what they have to say. If the professional notices the child avoiding a question or topic of discussion, it is best to leave it alone and circle back from another angle later on rather than repeat the same comment or question. Repeating the same comment or question can signal frustration to the child and reduce their comfort in the conversation and their trust in the provider. Another rapport-enhancing technique is to normalize any expression that might make the child embarrassed, including feelings of sadness, fear or anger. It is helpful to keep interactions with young children short (certainly no longer than one hour) and to ask questions made up of short simple sentences rather than compound or complex questions [4].

One technique for verifying that children understand the information received by providers is the “teach-back” method. This technique involves providing education to patients and families then asking them to paraphrase what they have just learned. The “teach-back” process has been shown to improve understanding while simultaneously addressing health literacy [19]. Children reluctant to “teach-back” may be encouraged by using “What” and “How” questions, expressing empathy to the child, repeating a statement the child made and then pausing, making an open-ended statement or question, and providing the child with praise for communicating [4].

Young children, and some older children and adults, such as some individuals on the Autism spectrum, are concrete thinkers. Concrete thinkers have difficulty understanding, reflecting on and communicating about ideas and concepts. They are more likely to share thoughts about actions, objects, events, and people. They are more likely to use the literal rather than the figurative meaning of words and phrases. Professionals (and many caregivers) are frequently unaware of the abstract nature of their communications. They must be intentional in their use of concrete language, and use verbs as a way to reformulate abstract concepts into action-based language [4]. For example, a teen on the Autism spectrum might have a fearful response when informed that the team is going to evaluate her headaches by taking a look at her brain, not realizing that the team plans on ordering an imaging study, not brain surgery.

It is helpful for clinicians to understand that young children have difficulty with chronology and time perception. This can become important when attempting to determine the best time to inform a child of an upcoming procedure or other painful event. Provision of accurate procedural information should account both for child age and time to procedure. Children six and above who are given information about their surgery 5 to 7 days prior tend to show a reduction in pre-procedural anxiety [20]. By contrast, children younger than three cannot effectively make use of preparatory coping resources and are thus not as aided by advanced warning of planned surgeries [20]. Young children are also limited in their ability to imagine future physiological states and to anticipate their needs for the forthcoming occasion [21,22]. They are often unable to concentrate on a future need (e.g., need for comfort) if they are preoccupied with a different desire at present (e.g., need to quench thirst) [23]. Children younger than six should thus be told closer to the surgical date [24].

It is both helpful and unhelpful that young children try to please adults. On the one hand, they might be more willing than a teenager to engage in discussion with a professional, but they may also be more unlikely to inform the professional if they are confused. This indicates the importance of using the teach-back method to confirm understanding. Signs of confusion during a clinical interview include a pattern of answers such as repeated “Yes/No,” “Maybe,” “I’m not sure,” “I guess,” “A little,” and “Because” answers, and repeatedly confirming the last option in a series that the interviewer presents in a question. If the conversation suddenly seems derailed by the child, it can be helpful to determine if the child is trying to provide the professional with important information, is avoiding the topic, or has not understood a question [4].

If a child suddenly shuts down communication, the professional might benefit from tracing back the conversation to the moment that the behavior of the child changed. Some children enjoy being asked to help the professional understand. Some are unable to articulate why they suddenly feel unable or unwilling to continue the discussion. Problems with understanding, emotional reactions or privacy issues should be considered. For example, even young children may wish to protect their caregivers by not sharing their fears about pain or desire to have (or not have) certain medications.

It is important that the professional take care not to ask leading questions while communicating with a young child. With a strong desire to please adults, some children will focus more on accepting information and responding in a pleasing manner than reflecting on the information and providing honest reactions and answers. This means information and questions are best delivered in a neutral manner [4].

The communication techniques described above can be helpful when communicating with tweens and adolescents. However, additional developmental considerations are important as youth enter and transition through adolescence. It is helpful to remember the developmental goals of teens in order to most effectively communicate with them. Adolescents are preparing for adulthood, requiring practice in independent and joint decision-making, expression of their unique perspectives, and individuation from their caregivers. As with younger children, rapport can be enhanced by discussing a common interest, creating a fun interaction, and by listening to them, especially about their past experiences and concerns related to pain. It is particularly important to ensure that teens have a private place to share information apart from their parents if desired. It is advisable to avoid providing mini-lectures or mandates that might provoke a developmentally expected opposite behavior. Developing pain assessment or treatment plans jointly with the teen will generally yield better cooperation. Finally, adolescents who are typically developing may find benefit via the use of pain analogies, described below [4].

## 5. Youth with Developmental Disabilities

Youth with developmental disabilities are significantly more likely to have comorbid chronic medical conditions that require frequent healthcare interventions [25]. Certain developmental disabilities are associated with a higher prevalence of pain conditions and/or sensory sensitivity. As examples, cerebral palsy is associated with a high prevalence of functionally limiting pain [26,27]; Autism is associated with sensory sensitivity as well as social communication challenges [4]. Given the comorbidities between developmental disabilities and healthcare utilization that may involve pain, it is crucial for providers to understand how to alter information provision and preparation for youth with pain and developmental disabilities.

Children and adolescents with developmental disabilities may have a particularly difficult time describing pain due to the abstract language that it typically requires. For children with limited expressive language, nonverbal expressions of pain may be more common. Moreover, social cognition, which is necessary for children to grasp the need to express pain in order to get help, may be difficult for children with developmental delays due to limited theory of mind. Research shows that children with developmental disabilities display fewer nonverbal pain communication cues (facial grimacing and crying) than children without delays [28]. They also appear less likely to seek assistance from adults when in pain [28]. Children with developmental disorders such as Autism Spectrum Disorder may experience atypical sensations and demonstrate uncommon expressions of pain [29]. Importantly, research has also shown that individuals with Autism may recover more slowly from acute pain experiences (e.g., venipuncture) [30]. The above findings serve to correct the misconception that children with developmental disabilities may be less responsive to pain, instead highlighting the social skills deficits that inhibit accurate social communication about noxious stimuli [28].

The discrepancy between pain sensation and pain communication in children with developmental disabilities highlights the importance of reliably assessing pain in these children. Proxy reporting of pain by caregivers and nurses has been shown to often be inaccurate [31]. Instead, observing changes in behavior may be a more consistent metric of discomfort in children with communication difficulties [12]. The use of augmentative and alternative communication (AAC) systems presents an additional tool for fostering pain communication in children with limited expressive language [12].

## 6. Types of Pain

Strategies to aid youth in preparing for pain related to medical intervention differ from those that seek to help youth with pain that has become chronic.

### 6.1. Acute

To aid in coping with acute pain associated with routine procedures, caregivers are urged to accurately describe the event ahead of time and to be forthright about the anticipated discomfort [32,33]. Venipunctures (whether for vaccines or blood draws) are one frequent source of anxiety in routine medical care. Most children fear needles though prevalence of needle phobia decreases with age [34]. Having caregivers announce the upcoming venipuncture a few days ahead of the procedure is helpful. Advance notice of the appointment helps avoid a spike in anxiety upon arrival and minimizes distrust that may stem from feeling ”swindled” into medical care by the caregiver. Caregivers should avoid promises that shots will be painless. Instead, caregivers should emphasize that, while there may be stinging, the pain will be brief. In younger children, the use of breastfeeding or the administration of a sweet-tasting solution (e.g., sweetened water) prior to and after the injection has been shown to minimize discomfort [35]. Informing children about forthcoming medical procedures using accurate descriptions has been associated with lower peri-procedural distress and better adjustment [2]. Topical anesthetics, applied to the area up to an hour before the shot, can also minimize pain [35]. The use of distraction is always recommended [36]. Caregiver reassurance (e.g., “It’s going to be ok.”) does not appear to be useful and may, in fact, contribute to child distress [37]. Parents should be prepared to help their child focus on something else other than the procedure (toys, books, videos, games etc.) [38]. Finally, sitting younger children in caregivers’ lap while being hugged also has utility for providing comfort and for keeping the child still [35]. After the procedure, emphasizing what went well (e.g., bravery, compliance) can solidify the memory of the event as routine rather than as traumatic [33].

Pain related to medical treatment can be particularly distressing for children. Parents can be important allies to medical professionals by preparing children for procedures using honest, developmentally-tailored descriptions that will allow children to plan for the event. For more serious medical procedures, descriptions of the event are essential. While the extent of the information will vary based on the child’s age, children should be told that they will be going to the hospital (“for an operation”, for example). A brief explanation should be understood by most children though keeping descriptions brief and concrete is recommended [39]. For caregivers uncertain about what to say to their child, involving child life specialists can be helpful. These providers are experts in explaining procedures using developmentally-tailored language. Their involvement in medical settings has also been associated with decreased distress in children undergoing procedures [40].

As mentioned earlier, children six and older should receive preparatory information about medical procedures at least five days in advance [20]. Children should be told what the hospital will be like and what they can expect before, during, and after the procedure [20].

Providers should include both sensory (explanation of anticipated feelings and sensations) and procedural (what will be done, where, in which part of the hospital) information. Written materials are not enough [24]. Instead, use of dolls for role-plays and tours of the surgical units can be helpful in preoperative preparation as they allow children to imagine the scenario and bolster coping skills in advance [41].

Prior to the procedure, caregivers can help by engaging their child in play activities that are familiar, comforting, and distracting (reading a favorite book, singing a song, telling a story, blowing bubbles, playing a video game, etc.) [20,35]. During procedures, caregivers and healthcare providers should avoid reassurance, criticism, and apology as these have been shown to cause more distress and poorer coping [1]. Similarly, while giving children choices appears to support coping, giving children control over the procedure tends to cause more distress [1].

Young children can be told about the procedure the day before, middle-aged children and teens can be told a few days in advance. Too much notice for young children may be more anxiety provoking than necessary [20,24]. Finally, it is crucial to emphasize that caregiver communication directly impacts child coping with pain. In particular, children whose caregivers used invalidating language and behavior report more pain and distress [42]. Parents who rely on their presence alone instead of distraction also tend to have children who are more distressed [43].

For children undergoing anesthesia, additional language may be necessary. The American Academy of Anesthesiologists recommends preparing children by telling them that they will take a nap after receiving medication that will ensure that they feel no pain during the procedure. Language that parallels deaths (e.g., being put to sleep) should be avoided [39]. Parents can also inform children that they may wake up from the procedure feeling groggy and confused but that this is normal and will resolve [44].

### 6.2. Chronic

Psychoeducation about chronic pain is a cornerstone of effective treatment and rehabilitation efforts. Pain neuroscience education (PNE) is a term that encompasses information for patients and families about the mechanisms that create and sustain chronic pain in the absence of acute tissue damage. Provision of PNE is an essential part of chronic pain treatment, especially for patients whose pain appears to be centralized as well as for those with maladaptive beliefs about pain (e.g., catastrophizing the effects of pain such that it harmful and damaging). PNE provides a common language between patients and providers that enables a proactive dialogue about ways to both cope with pain and function despite it [2].

Based on a biopsychosocial model of pain, PNE emphasizes that pain is affected by all spheres of a person’s life. PNE emphasizes that chronic pain can be affected by environmental contexts and does not necessarily require the presence of a noxious stimuli. PNE should include an explanation of the human tendency to a) identify a cause for our pain, and b) find ways to avoid it. One model to describe pain-related avoidance is the Fear Avoidance Model, which has been validated as helpful for pediatric patients and their parents [45]. Patients must understand that pain-related fear is a normal response to the experience of noxious stimuli and naturally tends to lead to avoidant behaviors aimed at reducing the re-experiencing of pain. However, too much fear avoidance is also problematic as patients with high fear avoidance are more likely to be disabled by their pain [45]. Providing patients with this education helps break the vicious cycle of pain-related avoidance and turns the focus towards functioning and quality of life improvement. It also shifts the focus away from the impossible task of preventing future pain [2]. The above education can be simplified depending on the patient’s developmental level. It can and should also be delivered to parents who may also fall victim to fear avoidance [45].

Analogies and metaphors are particularly useful in describing chronic pain. The use of allegorical language can help patients understand the mechanisms of chronic pain and the need to continue functioning despite pain. Pain is an evolutionary communication tool that allows our bodies to signal danger. Protective in nature, it allows us to attend to a wound or illness that may ultimately prove fatal. Pain is also a way to communicate our need for safety and/or for a change of course in our actions [46]. In chronic pain, the body continues to signal alarm despite a lack of organic cause of nociception [47]. Even if stemming from an injury, its signal may grow disproportionately to the pain associated with the original wound [46]. Helping patients understand this change in signaling from protective to maladaptive can be therapeutic for those who have experienced long-term pain and may be chasing an organic etiology that leads to healthcare overutilization. One analogy that may be useful is the description of chronic pain as a broken alarm system [47]. While the original injury or illness that dictated the need for an alarm is gone, the alarm is continuing to alert its host. For instance, chronic pain is like a damaged smoke alarm that goes off despite there being no smoke or fire in the house. Having a better understanding of the mechanisms that sustain chronic pain has been shown to decrease catastrophizing thoughts in patients with chronic pain [48].

Analogies of chronic pain may also be therapeutic for encouraging patients to distract themselves from the nociceptive input. If the alarm system has stopped being useful in that it no longer signals danger, it can and should be ignored. Given that pain is impacted by our focus on it, patients can learn to focus on daily functioning rather than on the sensations they are feeling. Comparing chronic pain to a creaky old house, for instance, can emphasize the need to ignore the “noises” of the body and instead continue carrying out daily tasks and living full, meaningful lives [46]. As chronic pain patients shift attention from the experiential intensity and frequency of pain to that of functional capacity (being able to go to school, enjoy friends, play games), chronic pain may lessen as a result of receiving less attention. Patients will also find emotional relief in being able to return to normal functioning after months or even years of prioritizing pain over daily activities [48].

Another important take away for patients is the idea that pain begets more pain. The experience of pain in the body sensitizes our nervous system to its signaling [2,49]. Like squirming away in anticipation of a tickle, the body can react and reproduce nociception when it expects it. As such, the brain that expects pain may in fact experience more pain. Patients that have had more painful experiences may have a brain sensitized to the over identification of pain signals [2,49]. Finally, chronic pain early in life can predispose the brain to developing chronic maladaptive pain signaling through changes in neural wiring [49].

## 7. Conclusions

Traumatic pain experiences in youth can lead to poor coping, loss of functioning, and long-term changes in neural pain centers. Such youth can also develop symptoms of medical trauma and be at higher risk for medical nonadherence. Healthcare providers and caregivers can minimize the risk for longstanding sequelae through the provision of developmentally-tailored, factual information, and through the encouragement of healthy, action-focused coping styles. In order to accurately assess and treat pain, healthcare providers should be educated about ways in which pain communication evolves in both neurotypical youth and those with developmental disabilities. Evidence-informed pain communication strategies to optimize coping differ for acute, procedural, and chronic pain. Acute and procedural pain require patient education, truthful information, and advance preparation. By contrast, the use of analogies is particularly helpful for patient understanding of chronic pain development, maintenance, and for its eventual treatment.

While research in this domain has elucidated the benefits of pain information provision, little is known about the ways in which such education impacts the medical course of patients with acute and chronic pain. Future research should focus on ways to more systematically examine the effectiveness of communication guidance on patient outcomes. Longitudinal studies that assess how developmentally-tailored pain education impacts patients’ trajectories and their healthcare utilization are warranted.

## Figures and Tables

**Table 1:** Piagetian Stages of Cognitive Development and the Experience of Pain [4].

Developmental Stage	Typical Implications Related to Pain
Sensorimotor children (birth to about 2 years old)	They are mostly preverbal, without the capacity to create narratives to explain their experiences. They are most likely to demonstrate pain by social withdrawal or changes in their patterns of sleep, eating, and level of activity. By 18 months of age, most typically developing children will make an effort to localize pain and seek reassurance from adults; 2-year-old children are often able to use specific words to indicate the presence of pain.
Preoperational children (about 2–7 years old)	They use words and understand basic concepts of cause and effect. However, they tend to erroneously see events that are temporally related as causally related. They may view pain as a punishment for the real or imagined transgression of rules. During this stage, some of the younger children are not able to use self-generated coping strategies and tend to rely on their environment (i.e., the support of adults). Children older than 3 might have this capacity. Children in this developmental stage have difficulty using rating scales of pain. They also have difficulty differentiating pain from anxiety or fear.
Concrete operational children (about 7–11 years old)	They can apply logic to their perceptions in a more integrative manner. However, the logic is literal (concrete) and allows for only one cause for an effect. Interventions that are concrete will make more sense to children at this stage. For example, applying a topical anesthetic to a painful part will make more sense to them than pain relief via oral or IV medication. At this stage, they are likely to be able to use a rating scale for pain assessment, and they will have an increased ability to use self-initiated coping strategies such as distraction or guided imagery.
Formal operational children (11+ years old)	They are able to use abstract reasoning to discuss body systems and can conceptualize multiple causation of pain. They are potentially more aware of the psychological aspects of pain and better able to understand a biopsychosocial model. Their greater ability to focus on future events may lead to greater worries and concerns about the pain. It should not be assumed that all adolescents (or caregivers) can utilize abstract reasoning. Most adults engage in abstract reasoning only in areas of their own expertise, if at all, everyone tends to regress under stress.

Caplan and Bursch, 2012, page 262; reproduced by permission of Oxford University Press, https://global.oup.com/.
